# Shaping the Immune Response: Cathepsins in Virus-Dendritic Cell Interactions

**DOI:** 10.3390/cells14231900

**Published:** 2025-12-01

**Authors:** Adrianna Niedzielska, Magdalena Bossowska-Nowicka, Zuzanna Biernacka, Karolina Gregorczyk-Zboroch, Felix N. Toka, Lidia Szulc-Dąbrowska

**Affiliations:** 1 Department of Preclinical Sciences, Institute of Veterinary Medicine, Warsaw University of Life Sciences-SGGW, 02-786 Warsaw, Poland; adrianna_niedzielska@sggw.edu.pl (A.N.); zuzanna_biernacka@sggw.edu.pl (Z.B.); karolina_gregorczyk-zboroch@sggw.edu.pl (K.G.-Z.); ftoka@rossvet.edu.kn (F.N.T.); 2 Institute of Animal Reproduction and Food Research of Polish Academy of Sciences, 10-683 Olsztyn, Poland; m.bossowska@pan.olsztyn.pl; 3 One Health Center for Zoonoses and Tropical Infectious Diseases, Ross University School of Veterinary Medicine, Basseterre P.O. Box 334, Saint Kitts and Nevis

**Keywords:** cathepsins, cysteine cathepsins, dendritic cells, DC, viral immune evasion, antigen presentation, DC-virus interaction, endolysosomal proteases

## Abstract

Dendritic cells (DCs) are among the first immune cells to detect viral invasion and play a central role in initiating and shaping antiviral immune responses. Many innate and adaptive immune functions of DCs are regulated by cathepsins, proteolytic enzymes primarily found in acidic endolysosomal compartments. Different DC subsets exhibit distinct cathepsin expression patterns, influencing their functional capacities and interactions with viruses. In DCs, cathepsins contribute to virus sensing through innate receptors, regulate cytokine production and DC migration, and are essential for viral antigen degradation and loading onto MHC molecules for T-cell activation. Many viruses, however, have evolved mechanisms to alter cathepsin expression and activity, thereby subverting DC function and promoting their own persistence. Indeed, cathepsins can facilitate viral entry into DCs, promote viral replication, and support immune evasion strategies. In this review, we summarize recent advances in understanding the role of cathepsins in DC–virus interactions, emphasizing both how DCs exploit cathepsins to generate protective immune responses and how viruses manipulate cathepsin activity to their advantage. We particularly focus on clinically relevant viral pathogens, including HIV, influenza virus, hepatitis C virus, human cytomegalovirus, Ebola virus, and SARS-CoV-2, to illustrate the multifaceted influence of cathepsins on DC biology during viral infection.

## 1. Overview of DC Subsets and Cathepsin Biology

**Figure 1:**
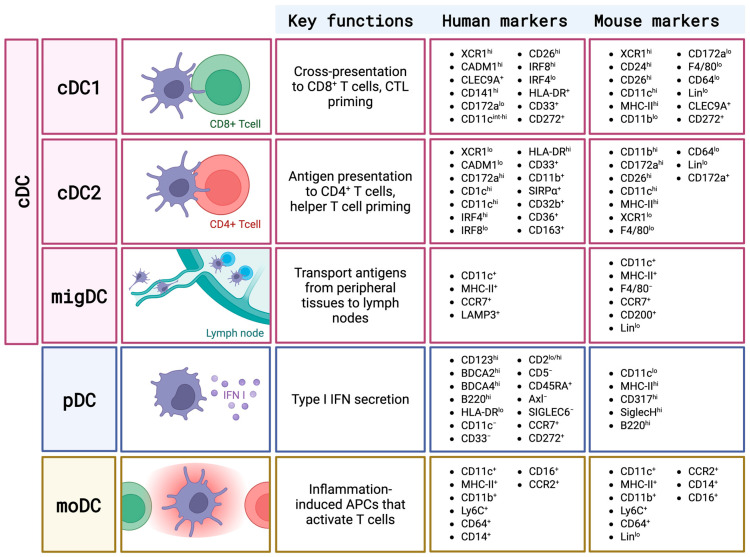
DC subsets and their key markers and functions. APC, antigen-presenting cell; cDC, conventional dendritic cells; CTL, cytotoxic T lymphocyte; IFN, interferon; migDC, migratory DC; moDC, monocyte-derived DC; pDC, plasmacytoid DC. Created in BioRender. Niedzielska, A. (2025) https://BioRender.com/56nrpqh (accessed on 26 November 2025) https://BioRender.com/56nrpqh.

Dendritic cells (DCs) regulate immune responses against various pathogens, including viruses, linking innate and adaptive immunity. By sensing pathogens through pattern recognition receptors (PRRs), DCs initiate signaling cascades that shape host defenses. Beyond maintaining immune homeostasis and tolerance, DCs act as key antigen-presenting cells (APCs) orchestrating antiviral immunity [1]. DCs are heterogeneous, differing by origin, tissue localization, receptor expression, and function, and are classified into conventional/classical DCs (cDCs), plasmacytoid DCs (pDCs), and monocyte-derived DCs (moDCs) [2,3]. cDCs, the largest and most diverse group, are subdivided into cDC1 and cDC2 subsets, and further into lymphoid-tissue-resident (LT) and migratory non-lymphoid tissue (NLT) DCs (migDCs) [2,4], which transport peripheral antigens to lymph nodes for T cell priming. The cDC1 subset, arising from myeloid precursors, expresses X-C chemokine receptor 1 (XCR1) and CD8α (CD103 in tissues) and specializes in cross-presenting antigens on major histocompatibility (MHC) class I to prime CD8^+^ T cells, producing IL-12p70 to promote T helper (Th) type 1 responses [2,5]. cDC2s, also myeloid-derived, express CD11b and signal regulatory protein (SIRP)α and present antigens on MHC II to prime CD4^+^ T cells, with subsets (cDC2A and cDC2B) driving anti- or pro-inflammatory Th responses, including Th2, Th17, and T follicular helper (Tfh) cells; they may also produce type I interferons (IFNs) during viral infections [2,6]. NLT migDCs share cDC features like cross-presentation, XCR1 expression, and Toll-like receptor (TLR)3 responsiveness [4,6,7]. pDCs, lymphoid-derived, produce type I IFNs and can present antigens during viral infections, expressing blood dendritic cell antigen-2 (BDCA-2), BDCA-4, CD123 in humans, and CD11clo, B220^+^, Siglec-H^+^ in mice [8]. moDCs arise from monocytes during inflammation, bridging innate and adaptive immunity, and share markers with cDC2s but uniquely express CD64, CD88, and MerTK [2,7]. Key functions and markers of DC subsets are shown in Figure 1.

Many key aspects of the innate and adaptive properties of DC are regulated by cathepsins (Cts)—a family of serine (Cts A and G), aspartic (Cts D and E), and cysteine (Cts B, C, F, H, K, L, O, S, V, X(Z/P), and W) peptidases. These enzymes primarily act in acidic compartments (lysosomes, endosomes) but can localize to the cytoplasm, nucleus, and extracellular space [9]. While Cts B, H, L, C, X, V, and O are ubiquitously expressed, Cts K, S, E, and W vary by cell type and activation state [9,10]. Cathepsins are classified by structure, catalytic mechanism, and substrate. Serine Cts use a serine-histidine-aspartate triad [11], aspartic Cts have two domains with catalytic aspartates [12], and cysteine Cts are mostly monomeric except for tetrameric Cts C [13]. They are synthesized as inactive precursors with an N-terminal signal peptide directing them into the endoplasmic reticulum (ER), followed by propeptide cleavage, mannose-6-phosphate modification, and trafficking via the cation-dependent mannose-6-phosphate receptor (CD-MPR) pathway to late endosomes, where they are activated by pH-dependent dissociation and proteolytic processing [13,14,15].

DC subtypes express distinct cathepsins, with levels and activity influenced by activation state and tissue environment (Table 1). Immature DCs have low or inactive cathepsins, which are upregulated and activated upon maturation to promote antigen processing and MHC peptide loading [16,17]. Human cDCs generally show slightly lower expression of cathepsins B, L, and S than moDCs [18], and human and murine cDC1s express slightly less than cDC2s [19,20]. In the murine Mutu DC line, which resembles cDC1, CtsS activity decreases upon CpG (TLR9 agonist) maturation [21]. In splenic cDC1s, stimulation with lipopolysaccharide (LPS), CpG-B ODN1826, and poly I:C upregulates *CtsL* and *CtsS* to levels comparable or higher than thymic cDCs. In murine cDC1, CtsX activity is similar to cDC2 and increases in Mutu DCs after CpG via IL-6 [21,22]. Comparable levels of CtsA and functional CtsG are detected in human blood cDC1 [11,23], while CtsD mRNA is present in human blood myeloid DCs [24]. No specific data exist for CtsE in cDC1 (Table 1). This cathepsin profile supports efficient cross-presentation and controlled CD8^+^/CD4^+^ T-cell activation during inflammation. Meanwhile, cDC2s express slightly higher levels of cathepsins B, L, and S compared to cDC1s [19,20]. Human blood cDC2s also have higher levels of cathepsins involved in MHC II antigen processing, including X, H, and C [25]. In murine splenic cDC2s, CtsX activity is similar to cDC1 [22]. Serine cathepsins A and G are comparably expressed across human blood cDC2s and other DC subtypes [26], while aspartic CtsD is detected in human myeloid DCs, including cDC2s [11]. Other cathepsins (F, K, O, V, W) are generally low or negligible in expression. These profiles align with the cDC2 role in MHC II-restricted antigen processing. By analogy with cDC2, migDCs express moderate CtsX and baseline CtsH and C [21] (Table 1). pDCs have a distinct profile: CtsL and CtsS mRNA are higher in splenic/thymic pDCs than cDCs [16]; human pDCs show higher CtsB but lower CtsS compared to cDCs [24]. CtsX and H are low [24], whereas CtsC is high, with extracellular release upon influenza stimulation [27]. CtsV is relatively high in peripheral blood pDCs [24], CtsD mRNA exceeds myeloid DCs [24], and CtsE may be expressed at low levels [24]. This pattern supports viral sensing and type I IFN production. moDCs show the highest cathepsin expression among DCs, including CtsB, L, S, and X, with CtsX increasing upon LPS maturation [18,28]. CtsH [23] and CtsC (especially in tolerogenic moDCs) [29] are present. Comparable CtsA [30] and functional CtsG [11] are found across DC subsets. CtsD expression is regulated by peroxisome proliferator-activated receptor γ (PPARγ) [31], while CtsE is detected in human myeloid moDCs and murine BMDCs but remains unchanged with LPS [26]. CtsF, K, O, V, and W expression is low (Table 1). Robust cathepsin activity in moDCs underpins their potent proteolytic and antigen-presenting functions. In summary, the distinct cathepsin expression profiles across DC subtypes closely reflect their specialized roles in antigen processing and immune regulation.

## 2. Role of Cathepsins in the Function of DCs During Viral Infections

**Figure 2:**
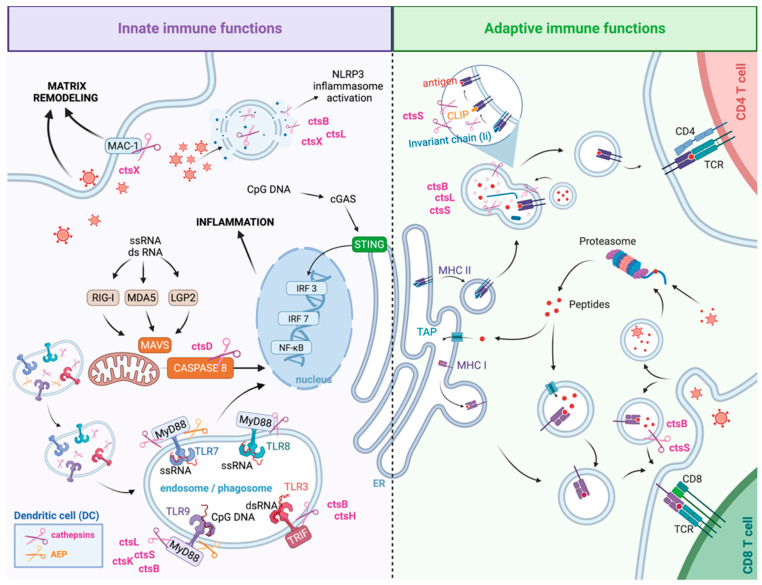
Role of cathepsins in innate and adaptive immune functions of DC during viral infections. Cathepsins regulate multiple aspects of DC biology in response to viral infection. In endosomes, cathepsins activate Toll-like receptors (TLR3, TLR7, TLR8, and TLR9) by proteolytic cleavage, allowing DCs to recognize viral nucleic acids and trigger signaling through MyD88 or TRIF. These pathways activate transcription factors such as IRF3, IRF7, and NF-κB, leading to the production of inflammatory and antiviral molecules. Within endosomal and phagosomal compartments, cathepsins together with AEP break down internalized viral proteins into peptides that are loaded onto MHC class II molecules and presented to CD4^+^ T cells. Cathepsins also process the invariant chain (Ii) and remove the CLIP peptide from MHC II, ensuring efficient antigen loading and T-cell priming. In the cytosol, viral DNA detection by cGAS activates STING supports antigen cross-presentation on MHC I molecules to CD8^+^ T cells. Cathepsins also contribute to DC maturation, extracellular matrix remodeling, and cytokine production, all of which are crucial for DC migration and the activation of adaptive immune responses during viral infection. AEP, asparagine endopeptidase; cGAS, cyclic GMP–AMP synthase; CLIP, class II-associated invariant chain peptide; DC, dendritic cell; dsRNA, double-stranded RNA; ER, endoplasmic reticulum; IRF, interferon regulatory factor; LGP2, laboratory of genetics and physiology 2; MAC-1, macrophage-1 antigen; MAVS, mitochondrial antiviral-signaling protein; MDA5, melanoma differentiation-associated protein 5; MHC, major histocompatibility complex; MyD88, myeloid differentiation primary response 88; NF-κB, nuclear factor kappa-light-chain-enhancer of activated B cells; ssRNA, single-stranded RNA; STING, stimulator of interferon genes; TAP, transporter associated with antigen processing; TCR, T cell receptor; TLR, Toll-like receptor; TRIF, TIR-domain-containing adapter-inducing interferon-β. Created in BioRender. Niedzielska, A. (2025) https://BioRender.com/jyawytg (accessed on 16 November 2025).

Cathepsins are key regulators of both innate and adaptive DC functions, coordinating immune responses from viral sensing to virus-specific T cell activation. They support innate immunity by activating endosomal TLRs (TLR3, TLR7, TLR8, TLR9), triggering antiviral signaling and type I IFN/pro-inflammatory cytokine release [21], and influence inflammasome activation, DC adhesion, and migration to ensure effective antigen presentation [28,32,33,34,35]. In adaptive immunity, cathepsins promote DC maturation and process viral proteins into peptides for MHC I and II presentation, shaping CD4^+^ and CD8^+^ T cell priming and polarization [36,37,38]. By regulating adhesion, migration, and antigen processing, cathepsins bridge innate antiviral defenses with long-term, antigen-specific immunity, representing a fundamental axis in viral infection pathogenesis that balances antiviral defense with potential tissue damage and inflammation (Figure 2).

### 2.1. Cathepsins in Innate Immune Functions of DCs

#### 2.1.1. Virus Sensing

DCs express the broadest and highest levels of PRRs to detect viral pathogen-associated molecular patterns (PAMPs) [39,40]. Endosomal TLRs recognizing viral nucleic acids, such as TLR3, TLR7, TLR8, and TLR9, undergo multistep proteolytic maturation, initially cleaved by cathepsins or asparagine endopeptidase (AEP), followed by secondary cathepsin trimming for optimal activation [41]. Resident and migratory cDC1 predominantly express TLR3, responding to dsRNA via the TIR-domain-containing adapter-inducing interferon-β (TRIF) adaptor pathway, which upregulates antiviral genes [42]. TLR3 detects viruses including rotavirus, respiratory syncytial virus (RSV), West Nile Virus (WNV), flaviviruses, Dengue virus (DENV), hepatitis B and C virus (HBV, HCV), herpesviruses, retroviruses, orthomyxoviruses, poxviruses, and certain strains of cytomegalovirus (CMV) [43,44,45,46]. In primary human moDCs, TLR3 exists as a full-length receptor and two lysosomal cathepsin B- and H-generated fragments [8]. TLR3 traffics through the ER–Golgi–endosome pathway, requiring UNC93B1 for endosomal localization. In acidic endosomes, cathepsin cleavage produces a stable C-terminal fragment essential for receptor activation, signaling, and retention [47].

Endosomal TLR7 and TLR8 recognize viral ssRNA, with TLR7 mainly expressed in pDCs and TLR8 in myeloid cDC2 and moDCs [48]. Both detect ssRNA viruses such as influenza, human immunodeficiency virus (HIV)-1, and coxsackievirus B [44,49,50]. TLR9, which is strongly and predominantly expressed in pDCs, senses CpG motifs in viral DNA from herpes simplex virus (HSV), CMV, Epstein–Barr virus (EBV), and vaccinia virus (VV), initiating antiviral immune responses with IFN type I production [51,52,53,54]. TLR7–TLR9 are synthesized as full-length proteins, transported from the ER to endosomes via UNC93B1-associated protein, and cleaved at the flexible loop region between leucine-rich repeats (LRR) 14 and 15 (Z-loop) for activation [55]. TLR7 is processed by furin-like convertases, AEP, and cathepsins, while TLR8 cleavage depends mainly on cathepsins [41,56]. Initial TLR9 processing in pDCs depends on AEP, which cleaves TLR9 into a C-terminal fragment that associates with myeloid differentiation primary response 88 (MyD88) for signaling. Although AEP also plays a role in the maturation of cathepsins B, H, and L [57], TLR9 activation remains functional without these proteases, indicating a dominant role of AEP. AEP directly cleaves TLR9 at a flexible loop in acidic endosomes, enabling CpG DNA binding and receptor activation. If AEP is absent, cathepsins can compensate for its function, though cathepsin-dependent trimming enhances signaling efficiency [41]. Conversely, when cathepsin activity is inhibited, AEP can still mediate the initial cleavage. However, without cathepsin-dependent trimming, the receptor remains in a slightly larger cleaved form, which leads to reduced signaling efficiency [41]. In macrophages, which have highly acidic compartments, TLR9 signaling proceeds independently of AEP. TLR7 undergoes similar proteolytic maturation [41,58].

Because TLR9 processing with cathepsins is cell-type-dependent, identifying the specific enzymes involved remains challenging and varies among immune cells. In BMDCs, inhibition or deficiency of cathepsin K impairs TLR9 signaling upon CpG DNA stimulation, reducing IL-12 production despite preserved antigen presentation capacity [59]. On the contrary, other studies report that cathepsin K deficiency does not affect TLR9 cleavage or signaling in BMDCs [60]. In macrophages, cathepsins L and S may also contribute to TLR9 processing, as their absence partially decreases TNF production in response to CpG DNA. Together, these findings indicate that cathepsin-mediated TLR9 processing is a finely tuned, cell-type-specific mechanism regulating the sensing of viral infections.

#### 2.1.2. Inflammatory Cytokine Production

Since cathepsins regulate the activation of endosomal TLRs in DCs, they directly influence downstream signaling that drives IFN and cytokine production. Cathepsins can either promote or limit cytokine output (Figure 2). For example, CtsB-deficient cDCs produce less IFN-α/β after modified vaccinia virus Ankara (MVA) infection [61]. CtsS enhances IL-6 production by splenic DCs and promotes Th17 differentiation following LPS exposure [62]. CtsD is released into the cytoplasm during poly I:C stimulation of DCs, where it interacts with IFN-β promoter stimulator 1 (IPS-1/MAVS) to promote caspase 8 cleavage and NF-κB activation [63]. CtsX supports TNF-α, IL-12p70, and IL-10 production in LPS-stimulated moDCs [28].

Certain cathepsins can also suppress cytokine production by DCs. Neutrophil-derived cathepsin G, released during hapten skin sensitization, inhibits IL-12 production by hapten-presenting DCs and limits CD4^+^ T cell differentiation into IFN-γ- and IL-17-producing cells [64]. BMDCs from *Ctsb*^−/−^ mice produce higher IL-12p70/p40 levels after *Leishmania major* infection than WT cells [65]. Conversely, several studies have shown that cathepsin deficiency (CtsB, CtsL, CtsS) does not affect TNF, IFN-β, IL-6, or IL-10 secretion by BMDCs in response to LPS, CpG, live or killed *L. major* promastigotes, or ectromelia virus (ECTV) [65,66,67].

The relationship between cathepsins and cytokines is bidirectional, as inflammatory mediators also modulate cathepsin activity in DCs. TNF-α and IL-1β enhance CtsS and CtsB activity, promoting stable MHC II dimer formation [68]. IL-6 upregulates CtsX during CpG-driven DC maturation (and weakly with poly I:C) [21,69], whereas IL-10 suppresses CtsS and CtsB, thereby reducing DC antigen-degradation capacity [68].

Cathepsins also participate in inflammasome activation, particularly the NOD-, LRR- and pyrin domain-containing protein 3 (NLRP3) inflammasome, with CtsB, and to a lesser extent C, L, S, Z, and K, contributing to this process in DCs and macrophages [32,70]. NLRP3 provides a cytosolic platform for caspase-1 activation, processing pro-IL-1β and pro-IL-18 and inducing pyroptosis [71]. Viral infections can trigger lysosomal damage in DCs, releasing cathepsins (mainly CtsB, L, and Z) into the cytosol, where CtsB functions as an upstream NLRP3 activator [33,72]; influenza virus and coxsackievirus B3 use this CtsB-dependent mechanism to enhance pyroptosis [34,35]. Secreted CtsZ further boosts IL-1β production in BMDCs during NLRP3 activation via its integrin-binding motif [73]. CtsX is likewise essential for NLRP3-dependent IL-1β and IL-18 secretion, as *Ctsx*^−/−^ BMDCs show reduced cytokine release despite normal antigen presentation [74]. Loss of CtsX limits Th17 responses and neuroinflammation in the experimental autoimmune encephalomyelitis (EAE) mouse model of multiple sclerosis, and both CtsZ and CtsX are implicated in inflammation across cancer, silicosis, trauma, and chronic infections [69,74]. Overall, cathepsins can either amplify or restrain inflammasome-driven cytokine production, influencing the balance between antiviral defense and immunopathology.

#### 2.1.3. Adhesion and Migration

DC adhesion and migration, essential for antigen capture in tissues and subsequent T-cell activation in lymphoid organs, are also regulated by cathepsins. These proteases contribute to integrin regulation, extracellular matrix (ECM) remodeling, and DC movement through tissue barriers [28,75] (Figure 2). For example, CtsX activates the CD11b/CD18 (αMβ2, Mac-1) integrin in human moDCs by cleaving the C-terminal region of CD18, thereby enhancing adhesion to fibrinogen or plastic surfaces during maturation [28]. CtsE also supports DC adhesion and motility, as CtsE-deficient DCs show impaired migration in vitro and in vivo, which may contribute to reduced inflammation in graft versus host disease (GVHD) target organs after allogeneic hematopoietic stem cell transplantation in mice [75]. Inhibition of CtsX or CtsB disrupts podosome formation, key adhesive structures required for protease-dependent mesenchymal migration [28,76]. In contrast, CtsS deficiency does not impair lung DC migration to lymph nodes after LPS stimulation, indicating that CtsS is not required for DC-mediated antigen transport [77].

Cathepsins also influence DC motility by contributing to ECM remodeling through non-specific degradation of ECM proteins, occurring extracellularly or after endocytic uptake [78,79]. Such proteolysis supports immune cell migration and tissue remodeling during homeostasis and acute viral infection [80]. Extracellular release of cathepsins (e.g., B, L, S, K, V, X) typically occurs via vesicle-mediated exocytosis [78], and human DCs can secrete CtsD together with IL-1β through lysosomal exocytosis after interacting with alloreactive CD8^+^ T cells [81]. Although direct evidence remains limited, several observations suggest that DCs can release cathepsins during inflammation: DCs produce exosomes containing proteolytic enzymes [82]; lysosomal exocytosis occurs in related myeloid cells such as macrophages [83] and neutrophils [84]; and inflammatory stimuli promote lysosomal enzyme translocation to the DC plasma membrane [28]. Moreover, DC podosomes mediate localized ECM degradation via metalloproteinases, serine proteases, and cysteine cathepsins [85,86]. These findings highlight the likely, yet underexplored, contribution of extracellular cathepsins to DC function and immune regulation during viral infections.

### 2.2. Cathepsins and Adaptive Immune Functions of DCs

#### 2.2.1. DC Maturation

Cathepsin X is essential not only for DC migration but also for maturation by activating Mac-1 integrin [21,28] (Figure 2). During maturation, CtsX moves to the plasma membrane and later to the perinuclear region, aligning with reduced adhesion and acquisition of a mature phenotype. Inhibiting CtsX shifts DCs toward a tolerogenic state by lowering surface MHC II and co-stimulatory molecules (CD80, CD83, CD86) while increasing inhibitory Ig-like transcripts (ILT)3 and ILT4, thereby reducing cytokine production and T-cell stimulatory capacity [28]. CtsX expression during TLR9-dependent maturation is upregulated by IL-6 [21]. Conversely, CtsE-deficient DCs show increased CD86, CD80, and CD40 expression, resulting in enhanced T-cell activation [87].

CtsB, CtsL, and CtsS play only a minor role in regulating cDC surface maturation markers but are essential for antigen processing and presentation [88]. In murine JAWS II and BMDCs, CD80 and CD86 expression was similar in WT and CtsB-, CtsL-, or CtsS-deficient cells, regardless of maturation or stimulation with LPS, *L. major* antigen, or promastigotes, or ECTV [65,77,89]. Likewise, CtsB deficiency did not impair MHC II maturation, invariant chain degradation, or *L. major* antigen presentation [66]. Although some studies report higher MHC II levels in *Ctsb^−/−^* BMDCs after *L. major* exposure [65], this likely reflects parasite-driven modulation rather than a direct effect of cathepsin loss [90].

#### 2.2.2. Antigen Processing and Presentation

Cathepsins contribute to viral antigen processing for both MHC II and MHC I pathways, generating peptides from internalized proteins for CD4^+^ T cells and from endogenous proteins for CD8^+^ T cells [91]. Multiple cathepsins, including cysteine (B, F, H, L, S, X), aspartate (D, E), and serine (A, G), act within endosomal–lysosomal compartments, with CtsS being the key enzyme shaping the MHC II peptide pool in professional APCs such as DCs [36] (Figure 2). MHC II αβ heterodimers require the invariant chain (Ii) to stabilize their groove and guide trafficking from the ER through the trans Golgi network (TGN) to early endosomes, maturing into the MHC class II compartment (MIIC), where Ii and internalized antigens are degraded. After Ii cleavage, a class II-associated invariant chain peptide (CLIP) occupies the groove until exchanged by H-2M (mice) or HLA-DM (humans), allowing peptide loading and transport of mature MHC II–peptide complexes to the cell surface for presentation [92].

Studying cathepsin roles in antigen presentation is challenging, as in vitro findings often differ from in vivo outcomes. For example, CtsG cleaves recombinant Ii at neutral pH in vitro, but this may not occur in acidic endosomes [93]. Likewise, AEP inhibition delays CLIP expression and Ii degradation in vitro [94], yet AEP-deficient mice show normal Ii processing and MHC II maturation, indicating AEP is dispensable for antigen presentation in vivo but necessary for CtsL maturation [95].

CtsS is essential for Ii degradation and MHC II peptide loading, cleaving the p10 Ii fragment to form CLIP [96] and processing antigens such as myelin basic protein and proinsulin, the latter also aided by CtsD [97]. Highly expressed in DCs and spleen [98], distributed in endocytic compartments [38], and strongly inducible by IFN-γ [99,100], CtsS is a key enzyme for MHC II peptide generation [92]. CtsL also contributes to antigen processing but produces distinct peptide repertoires [101]. Although *Ctss^−/−^* DCs can still present MHC II/peptide complexes, mice show impaired immune responses to antigens administered with Freund’s adjuvant, including reduced antibody class switching and germinal center formation [102]. MHC II trafficking differs between immature and mature DCs, with mature cells relying more on recycled MHC II [103]. Pathogens modulate cathepsin-dependent processing: HCV reduces CtsS, impairing Ii degradation and altering MHC II loading [36], whereas influenza virus increases active CtsB, enhancing antigen presentation [104].

Cathepsins, especially CtsS, also support cross-presentation of exogenous antigens on MHC I in DCs. In the vacuolar pathway, CtsS generates peptides from internalized antigens, which are loaded onto intraphagosomal MHC I delivered via endocytosis or ER/Golgi vesicles [105]. Active under neutral-to-mildly acidic endosomal conditions, CtsS efficiently processes antigens like ovalbumin (OVA), influenza nucleoprotein (NP), and polymerase acidic (PA) protein, where other cathepsins (CtsB, CtsL, CtsD) are less effective [38,102,106]. DC phagosomes preferentially fuse with CtsS-rich vesicles to ensure processing before acidification [107]. Some antigens enter lysosome-like storage compartments, distinct from early endosomes and MIIC, lacking CtsS but containing CtsX, suggesting CtsX supports long-term storage and delayed cross-presentation [88]. During viral infections, cDC cross-presentation is enhanced via stimulator of interferon genes (STING)-mediated DC maturation and type I IFN-dependent upregulation of antigen-processing machinery, linking innate signaling to CD8^+^ T cell activation [108].

#### 2.2.3. Activation and Polarization of the Th Immune Response

Cathepsins influence CD4^+^ T cell activation and polarization by shaping the repertoire and abundance of viral peptides on MHC II. In *L. major* infection, CtsB-deficient BMDCs showed higher MHC II, increased IL-12, and stronger Th1 responses than WT or *Ctsl^−/−^* cells, indicating CtsB may increase susceptibility [65]. However, Rasid et al. [66] reported similar IFN-γ production in co-cultures of WT and *Ctsb^−/−^* BMDCs with T cells from infected mice. In vivo, CtsB inhibition shifted Th2 to Th1 responses during *L. major*, hepatitis B antigen, and OVA stimulation [109,110,111,112,113], whereas CtsL inhibition promoted Th2 responses and worsened disease [114]. In contrast, CtsB or CtsL knockdown did not affect T-cell priming or Th polarization during ECTV infection [89].

CtsS in moDCs is essential for T-cell activation during HCV infection and regulates Th1 responses in vivo [36]. *Ctss^−/−^* mice immunized with OVA in complete Freund’s adjuvant show impaired Th1 responses and germinal center formation, while Th2 development and IgE production remain intact [102]. In a murine model of experimental autoimmune *myasthenia gravis*, *Ctss^−/−^* mice immunized with the α146–162 from Torpedo californica (T-AChR) peptide failed to elicit peptide-specific T-cell proliferation or secrete cytokines including IFN-γ, IL-2, and IL-10 upon in vitro restimulation [77]. In chronic atopic dermatitis, CtsS overexpression promotes Th1 differentiation via proteinase-activated receptor-2 (PAR-2), whereas its inhibition favors Th17 responses in perivascular adipose tissue after vascular injury in diabetic rats [36,115]. Overall, CtsS modulates DC-driven Th priming and polarization, affecting outcomes in viral infections.

## 3. Cathepsins as Targets of Virus Modulation in DCs

**Figure 3:**
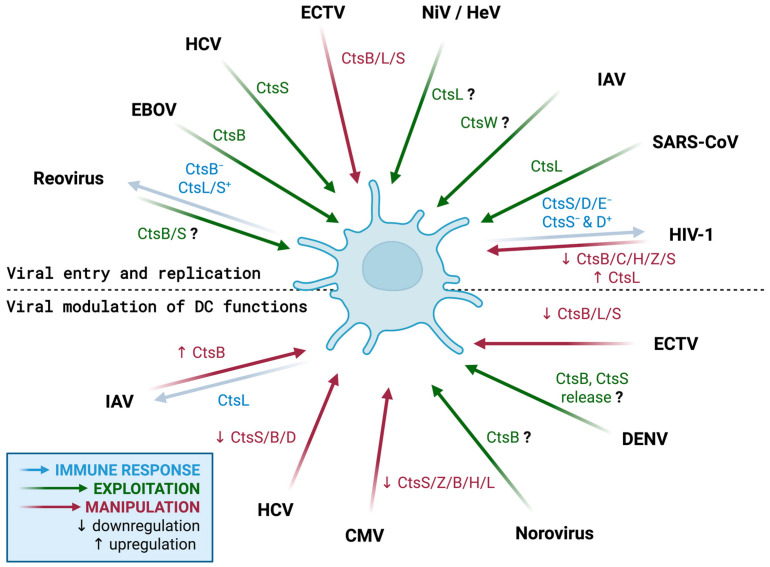
Cathepsins as targets of virus modulation in DCs. The blue arrow indicates cathepsins whose inhibitors enhance the immune response against the virus. The green arrow indicates cathepsins used by viruses to enter or replicate in the target cell. The red arrow indicates cathepsins whose levels are downregulated (downward arrow) or upregulated (upward arrow) by viruses during viral infection. Question marks denote steps that require further validation. EBOV, Ebola virus; NiV, Nipah virus; HeV, Hendra virus; SARS-CoV, severe acute respiratory syndrome coronavirus; HIV-1, human immunodeficiency virus type 1; IAV, influenza A virus; HCV, hepatitis C virus; CMV, cytomegalovirus; ECTV, ectromelia virus. Created in BioRender. Niedzielska, A. (2025) https://BioRender.com/gptm5k8 (accessed on 26 November 2025).

Besides an important role of cathepsins in regulating the innate and adaptive immune functions of DCs, which are essential for mounting an effective antiviral immune response, cathepsins may also be exploited by viruses to promote different steps of their replication cycle, including entry, uncoating, replication, assembly, or egress from DCs. Furthermore, the influence of viruses on the expression or activity of cathepsins in DC subsets may determine whether DCs effectively prime antiviral immunity or inadvertently facilitate viral spread, therefore affecting the balance between viral clearance and viral dissemination (Figure 3). Lysosomal remodeling in DCs has been shown to govern antigen processing, motility, and immune signaling, reinforcing the critical role of cathepsins in these cells [116]. Understanding the dual role of cathepsins in virus–DC interactions is therefore crucial for uncovering the mechanisms of immune defense and viral evasion.

### 3.1. Cathepsins in the Viral Replication Cycle in DCs

#### 3.1.1. Cathepsins in Viral Entry into DCs

The most studied function of cathepsins in the viral life-cycle involves their role in entry into target cells [117]. Viral entry, encompassing attachment, penetration, uncoating, and genome activation, is crucial for initiating infection and determines viral pathogenicity and cell tropism. This is especially important in the context of DCs because their susceptibility to viral entry influences antiviral response initiation and the efficiency of virus-mediated immune modulation.

Reoviruses serve as key models for studying these processes in diverse cells, particularly cancer cells, where they preferentially replicate and exhibit strong oncolytic activity [118,119]. Myeloid DCs are also susceptible to reovirus infection [120] and are considered highly effective carriers for delivering oncolytic reoviruses to cancer patients [121]. The reovirus capsid consists of six structural proteins (σ1, σ2, σ3, μ1, λ1, λ2) forming two concentric layers, the inner and outer capsid. The outer layer typically contains 600 copies each of σ3 and μ1 [122]. Reoviruses attach to the junctional adhesion molecule-A (JAM-A) via the σ1 protein and enter cells through β1 integrin-dependent endocytosis [123]. JAM-A also has a broader immunological role, since it functions as a positive regulator of Th1 differentiation by facilitating interactions between CD4^+^ T cells and DCs during T cell priming [124]. In DCs, however, reoviruses engage alternative targets such as sialic acid residues [121]. Within endosomes of fibroblasts, cathepsins B and L remove σ3 and μ1 to form infectious subvirion particles (ISVPs) [125], while in macrophage-like cells, this process is mediated by cathepsin S [126]. Given the high expression of cathepsin S in various DC subsets, this enzyme may similarly facilitate reovirus entry into DCs.

Ebola virus (EBOV), a filovirus, also exploits cathepsins to enter DCs and macrophages, which are the first cells at mucous membranes to encounter the virus [127]. EBOV preferentially infects CD11b^+^ cDCs (cDC2) and moDCs, whereas the migratory cross-presenting CD103^+^ DCs are largely spared. Infected DCs may transport EBOV from the entry site to draining lymph nodes, acting as key portals for viral dissemination and viraemia [127]. EBOV enters the DC cytoplasm via its envelope glycoprotein (GP), composed of GP1 and GP2, which mediate membrane fusion. N-linked glycans on GP1 bind C-type lectins on DCs, including DC-SIGN, L-SIGN, hMGL, and LSECtin, facilitating macropinocytic internalization into early endosomes [128,129]. In late endosomes/lysosomes, cathepsins B and L proteolytically process GP1, removing the mucin-like domain and glycan cap (~60% of GP1) and generating shorter subunits. This processing enables GP1 to bind Niemann-Pick type C1 (NPC1), triggering GP2-mediated membrane fusion and cytoplasmic entry [130]. In DCs, CtsB, but not CtsL, is essential for viral-like particle (VLP) entry, contrasting with Vero cells, where both, especially CtsL, are critical [131]. TNF-α modulates CtsB activity, with short treatments enhancing and prolonged treatments reducing entry [131]. EBOV glycoprotein processing varies by virus strain and cell type: Ebola-Zaire (ZEBOV) and Ebola-Côte d’Ivoire (CIEBOV) entry is strictly CtsB-dependent, whereas Ebola-Sudan (SEBOV) and Ebola-Reston (REBOV) are less dependent [132,133].

Certain viruses in the *Paramyxoviridae* family, including Nipah virus (NiV) [134,135] and Hendra virus (HeV) [136], enter cells via cathepsin-dependent mechanisms. Although these studies were performed in non-immune cells, both viruses can infect and replicate in DCs [137,138], suggesting that similar entry pathways may operate in these cells. In Vero cells, cathepsin L cleaves the NiV fusion (F) protein, enabling conformational changes required for membrane fusion and viral entry [135]. Inhibition or knockdown of CtsL blocks F-protein processing and membrane fusion, while *Ctsl^−/−^* fibroblasts lose fusion activity, restored only upon CtsL expression [135]. HeV similarly relies on CtsL for F-protein maturation and membrane fusion [136]. CtsL inhibition or shRNA silencing abolishes this process [136]. Although both F proteins can be cleaved by cathepsin B in vitro, such processing yields unstable, non-fusogenic products [135,136]. Collectively, these findings highlight the critical role of cathepsin L in NiV and HeV entry and suggest that cathepsin inhibitors warrant evaluation as potential antivirals.

Severe acute respiratory syndrome coronavirus 2 (SARS-CoV-2), a betacoronavirus, can also infect DCs and utilizes cathepsins, particularly CtsL, for host cell entry [139,140,141,142]. SARS-CoV-2 primarily infects epithelial cells expressing the angiotensin-converting enzyme 2 (ACE2) receptor and, possibly, DCs upon reaching the respiratory tract [143]. The interaction of SARS-CoV-2 with ACE2 is mediated by the viral spike (S) protein, which, together with the envelope (E), membrane (M), nucleocapsid (N), and accessory proteins, is essential for viral entry, assembly, and replication [144]. The S protein consists of two domains: S1, responsible for ACE2 binding, and S2, which mediates membrane fusion. SARS-CoV-2 entry into host cells can occur via two distinct pathways depending on the accessibility of cellular proteases required for S protein activation. In the first pathway, the virus is internalized by endocytosis, and the S protein is cleaved by CtsL in endosomes, enabling fusion of viral and endosomal membranes and release of viral RNA into the cytosol. In the second pathway, if transmembrane serine proteases (TTSPs), such as transmembrane serine protease 2 (TMPRSS2), are present on the cell surface alongside the ACE2 receptor, S protein cleavage at the S2′ site allows direct plasma membrane fusion, independent of endocytosis [144]. Because TMPRSS2 expression is lower in cDCs and pDCs than in epithelial cells [145], SARS-CoV-2 is likely internalized via clathrin-mediated endocytosis. Within endosomes, CtsL cleaves S1, exposing the fusion peptide and promoting fusion of viral and endosomal membranes, releasing the viral RNA into the cytosol [146]. Interestingly, SARS-CoV-2 spike protein evolves over time to alter its preference for host proteases, enabling variant-specific entry through diverse TTSPs beyond TMPRSS2. For example, the Omicron (BA.1) variant favors cathepsin-dependent endosomal entry due to less efficient S1/S2 cleavage, whereas earlier variants (Wuhan, Delta) primarily utilize TMPRSS2-mediated plasma membrane fusion [147]. Such evolution of the SARS-CoV-2 spike cleavage site enhances transmissibility, alters cell tropism, and can influence disease severity and transmissibility. It cannot be excluded that Omicron adaptation to reduced S1/S2 cleavage and altered protease preference may allow it to exploit the entry pathway that is fully operative in DCs, potentially enabling more efficient infection of these cells compared with previous variants. Therefore, the precise contribution of cathepsins to SARS-CoV-2 entry into DCs should be determined in further investigation. Meanwhile, circulating CtsL levels may serve as an indicator of disease progression and severity in SARS-CoV-2 infection [91]. Higher CtsL expression is observed in head and neck squamous cell carcinoma (HNSCC) tissues and is associated with immune dysregulation, increased infiltration of multiple immune cells (including CD8^+^ T cells, CD4^+^ T cells, B cells, neutrophils, macrophages, and DCs), and greater susceptibility to SARS-CoV-2 infection [148]. CtsL inhibitors significantly reduce infection and represent a promising therapeutic approach for COVID-19 patients [142,149].

Influenza A virus (IAV), an orthomyxovirus, primarily replicates in respiratory epithelial cells by binding N-acetylneuraminic acid (sialic acid) through its hemagglutinin (HA) glycoprotein. However, IAV can also infect lung DCs [104]. Following attachment, the virus enters cells via clathrin-dependent or -independent endocytosis and macropinocytosis. Endosomal acidification then induces membrane fusion, releasing viral ribonucleoproteins (vRNPs) into the cytoplasm for nuclear transport and replication [149]. CtsW plays a key role in the late stages of IAV entry in fibroblasts, facilitating vRNP release from late endosomes. It’s knockdown traps vRNPs in endosomes and inhibits infection [150]. Consistently, *Ctsw^−/−^* mice exhibit reduced IAV susceptibility and increased survival compared with wild-type controls [117,151].

In contrast to their supportive role in viral entry in DCs, cathepsins can also exhibit inhibitory effects. CtsB has been shown to inhibit CD4-independent HIV-1 entry into host cells. HIV-1 enters cells either through the classical CD4-dependent route or via a CD4-independent pathway, in which the envelope glycoprotein gp120 binds directly to CCR5 or CXCR4. Both pathways promote productive infection of HIV-1 in DCs [152]. While CD4-dependent entry typically occurs through direct fusion at the plasma membrane, the CD4-independent route often depends on clathrin-mediated endocytosis and endosomal acidification, which activate cysteine cathepsins to facilitate viral translocation into the cytoplasm [153]. Interestingly, CtsD may modify the structure of gp120, increasing its affinity for co-receptors and promoting HIV-1 fusion at the plasma membrane [154]. Different DC subsets express CD4 along with the co-receptors CCR5 and CXCR4, all mediating HIV-1 binding and entry [155]. Yoshii et al. [156] demonstrated that inhibition of cathepsin B with the CA074Me inhibitor enhances CD4-independent HIV-1 infection in HeLa cells, whereas blocking endocytosis suppresses it without affecting CD4-dependent entry. Therefore, CtsB may act as a host defense factor, limiting infection of target cells by HIV-1. HIV-1 can also exploit DC-specific intercellular adhesion molecule-3, grabbing non-integrin (DC-SIGN) on DCs to attach and enter through a lectin-mediated route. Unlike the other mechanisms, DC-SIGN-mediated uptake primarily enables viral capture and subsequent transfer to CD4^+^ T cells, promoting trans-infection rather than productive replication within DCs [152]. Taken together, cathepsins have dual roles in viral entry into DCs and other cell types, functioning as both enhancers and inhibitors of infection, and their activity critically influences viral invasion and dissemination.

#### 3.1.2. Cathepsins in Viral Replication, Release, and Dissemination

Cathepsins may facilitate later stages of viral replication in dendritic cells, extending their role beyond initial entry. For influenza A virus (IAV), CtsB is required for optimal production of progeny virions in macrophages, as *Ctsb^−/−^* cells show reduced hemagglutinin (HA) expression and production of progeny virions. However, CtsB is not essential for viral RNA synthesis or virion incorporation, as demonstrated in *Ctsb^−/−^* macrophages and CA-074Me-treated A549 cells [157]. Thus, CtsB is important for HA expression and virion production but not for initial steps of viral entry or RNA replication. Notably, there is no direct experimental evidence that cathepsins are required for IAV replication in DCs. Instead, cathepsins have been implicated as targets for IAV-mediated modulation of DC functions.

Similarly, during HIV-1 infection, cathepsins influence viral persistence and replication within DCs. HIV-1 triggers rapid productive infection in activated T lymphocytes, whereas in macrophages, DCs, and astrocytes, infection is less productive but causes complex cellular changes [158,159]. In DCs, HIV-1 primarily enters via DC-SIGN-mediated endocytosis, which directs most virions to endocytic vesicles for cathepsin-mediated degradation, preventing productive infection. However, 5–10% of the virus survives and is transferred to T cells at the immunological synapse [152]. This viral persistence is linked to the virus’s ability to modulate cathepsin activity in DCs. HIV-1 exposure for 48 h alters lysosomal enzyme expression, downregulating cathepsins B, C, H, and Z, as well as cathepsin S RNA, while increasing cathepsin L expression and reducing cystatin C activity in moDCs. These changes collectively diminish cathepsin activity, impair viral degradation in late endosomes, and enhance survival of progeny virions, which can be re-endocytosed to facilitate DC-to-T cell transfer [158,159]. By redirecting the surviving virus through the immunological synapse, DCs inadvertently promote efficient transfer of HIV-1 to T cells, enhancing viral dissemination while potentially evading immune detection. Moreover, infected DCs and macrophages may amplify virus replication by processing viral antigens with CtsB [152], since CtsB is required for efficient release of HIV-1 Gag pseudoparticles, as its inhibition or deficiency in macrophages and HEK293T cells leads to accumulation of viral particles in autophagosomes and reduced extracellular release [160]. Additionally, CtsD secreted by vaginal epithelial cells enhances HIV transmission by promoting infection of CD4^+^ T cells and CD4-negative epithelial cells, with its activity influenced by hormones, semen, and the local microbiota. Cathepsin inhibitors (including cystatin CstB, Serpin A1, Serpin A3, and CstC) protect by blocking CtsD- and CtsB-mediated viral processing and replication [152].

HCV, a flavivirus, is also capable of productive replication in DCs, although the level of viral replication remains very low [161]. Similar to HIV, HCV exploits DC-SIGN on DCs to facilitate viral uptake and persistence. While HIV uses DC-SIGN to enter endosomes and partially evade degradation, allowing subsequent transfer to T cells via the immunological synapse, HCV binds its envelope glycoproteins E1 and E2 to DC-SIGN, promoting internalization into early endosomes where the virus is protected from degradation. This strategy likely allows HCV to avoid degradation by endosomal cathepsins because in early endosomes, the low-pH environment is not sufficient to fully activate these proteases. It is suggested that HCV may use DC-SIGN as a pathway to persist in DCs and enhance viral dissemination to hepatocytes [162]. Therefore, HCV, similar to HIV, uses this mechanism to survive within DCs and exploit them for dissemination to target cells.

Another example of a virus that evades cathepsin-mediated restriction to support its own replication in DCs is ectromelia virus (ECTV), an orthopoxvirus that serves as a model for poxvirus infections and pathogenesis in mice, closely resembling variola virus (VARV) infection in humans [163,164]. Infection of BMDCs with ECTV reduces mRNA expression and protein levels of cysteine cathepsins B, L, and S, as well as cystatins B and C, particularly in late stages of infection [89]. Cathepsin activity declines significantly, resulting in impaired endocytosis and processing of soluble antigens by infected BMDCs. Cathepsin L and cystatin B co-localize within viral replication centers from early infection, and siRNA-mediated knockdown of these cathepsins before infection enhances viral yields, indicating that ECTV manipulates cathepsins to optimize replication [89].

Cathepsins B, L, and S also differentially regulate reovirus pathogenesis. *Ctsb^−/−^* mice show improved survival, whereas *Ctsl^−/−^* and *Ctss^−/−^* mice exhibit reduced survival and impaired viral clearance due to defective cell-mediated immunity. While cathepsins are not required for establishing viremia, CtsL is essential for maximal viral replication in the brain. Pharmacologic inhibition of cathepsin L ameliorates disease and enhances survival by disrupting reovirus assembly, highlighting cathepsins as modulators of viral infection severity and potential therapeutic targets [118]. CD8α^−^/CD11b^lo^ and CD8α^+^/CD11b^lo^ DC subsets in the subepithelial dome (SED) of Peyer’s patches capture and process reoviral antigens from productively infected epithelial cells. Both subsets can present antigens via MHC II, inducing proliferation and IFN-γ production in antigen-specific CD4^+^ T cells [165]. Therefore, cathepsins not only regulate reovirus replication but also may influence DC-mediated functions, shaping adaptive immunity. Their central role in viral disassembly and pathogenesis makes cathepsins, particularly CtsL, attractive targets for therapeutic intervention, as pharmacologic inhibition can reduce viral replication and disease severity.

### 3.2. Cathepsins as a Target for Viral Modulation of DC Functions

#### 3.2.1. Cathepsins and Viral Modulation of Innate Immune Properties of DCs

Some viruses have evolved strategies to manipulate cathepsin-dependent PRR signaling in DCs, disrupting the detection of viral components and downstream immune activation. By interfering with pathways such as TLR signaling, some viruses can modulate proinflammatory cytokine and IFN I production, and programmed cell death induction in DCs [166,167,168,169]. In most cases, viruses inhibit PRR-signaling pathways by their direct interactions with adaptor proteins, PRRs, or downstream signaling molecules in DCs, thereby preventing the activation of transcription factors such as NF-κB, IRF3, and IRF7, and ultimately suppressing the production of type I IFN and proinflammatory cytokines [170]. On the other hand, some TLR-induced antiviral responses triggered by viruses may be beneficial to the virus and harmful to the host, suggesting an important role for cathepsin-dependent lysosomal proteolysis in the regulation of virus survival and dissemination [169,171]. For example, TLR3-mediated inflammation facilitates West Nile virus (WNV) entry into the central nervous system by disrupting the blood–brain barrier (BBB), leading to lethal encephalitis [172]. WNV and the related Usutu virus (USUV) can productively infect DCs, which cross the BBB to promote neuroinflammation, with DCs playing a more prominent role in USUV neuroinvasion than in WNV. [173]. Meanwhile, overexpression of cathepsins B, C, E, G, H, K, L, S, W, and Z is observed during respiratory syncytial virus (RSV)-triggered activation of retinoic acid-inducible gene-I (RIG-I)-like receptors in airways of infected mouse, suggesting that infiltrated immune cells (including DCs) could also contribute to these protease production in response to RSV [174]. Cathepsins can influence DC viral sensing during entry: reoviruses exploit cathepsin S to modulate DC activation and antigen uptake [121,126], while HIV-1 alters lysosomal cathepsins (downregulating B, C, H, Z, and S and upregulating L) to impair viral degradation and innate sensing [158,159].

Virus-induced dysregulation of cathepsins can influence multiple types of programmed cell death, potentially either limiting or enhancing viral replication and dissemination [175,176]. While cathepsins have been shown to regulate cell death in various cell types, their role in controlling DC death during viral infection remains largely unexplored. Current evidence only points to an indirect effect, revealing a significant gap in our understanding of how cathepsins contribute to cell-death-mediated antiviral immune responses in DCs. For instance, Dengue virus (DENV), a flavivirus, infects DCs [177] and has been shown to induce lysosomal membrane permeabilization in HepG2 cells, partially via reactive oxygen species (ROS), leading to the release of cathepsins B and S, which trigger apoptosis through caspase-9 and caspase-3 activation [178]. It is plausible that similar apoptosis-inducing pathways occur in DCs [177]. In addition, cathepsin B can act as an upstream activator of the intrinsic apoptotic pathway to prolong the replication window of noroviruses [179], which preferentially infect DCs [180]. Supporting this, transcriptomic analyses of peripheral blood mononuclear cells from COVID-19 patients revealed marked upregulation of cathepsins B and L, associated with increased apoptosis and autophagy [176,181]. Highly pathogenic human coronaviruses, including SARS-CoV, MERS-CoV, and SARS-CoV-2, not only suppress IFN-mediated antiviral responses but also induce extensive cell death and cytopathy, releasing large numbers of virions and facilitating viral spread [176]. Hepatitis B virus (HBV), a hepadnavirus, impairs autophagy in Huh7 cells via HBx, which disrupts lysosomal acidification, leading to reduced lysosomal degradative capacity and the accumulation of immature lysosomes, possibly through interaction with V-ATPase that affects its lysosomal targeting [182]. Clinical analyses of liver tissues from chronic HBV patients showed elevated immature CtsD levels [182]. Meanwhile, CtsB exacerbates Coxsackievirus B3-induced myocarditis by activating the inflammasome and promoting pyroptosis, a form of programmed cell death [35].

#### 3.2.2. Cathepsins and Viral Modulation of Adaptive Immune Properties of DCs

DCs are professional APCs with the highest capacity to prime naïve T cells, so it is not surprising that viruses have evolved diverse strategies to manipulate these cells and dampen adaptive immune responses, often targeting cathepsins as key regulators of antigen processing and presentation. Within DCs, HCV impairs immune functions partly by downregulating cathepsins, particularly CtsS. Recent work confirms that CtsS is dynamically regulated in DCs during maturation and inflammatory stimulation [79]. Human moDCs exposed to HCV show reduced CtsS levels, impaired Ii degradation, and increased surface HLA-DR–CD74 complexes. Consequently, CD74 accumulation disrupts MHC II maturation and antigen presentation, hindering T cell activation [36]. In IFN-γ-treated hepatocytes, HCV core and NS5A nonstructural proteins suppress CtsS by inhibiting the transcription factors that regulate cathepsin S expression, i.e., IRF-1 and upstream stimulatory factor 1 (USF-1) [36]. Recent studies indicate that HCV NS5A also interacts with HSC70 and recruits it to diacylglycerol O-acyltransferase 1 (DGAT1), thereby inducing lysosomal degradation of DGAT1 via endosomal microautophagy [183]. Inhibition of DGAT1 has been shown to impair lysosomal homeostasis and displace cathepsin-positive endolysosomes, which may secondarily disturb cathepsin maturation and stability [184]. HCV-treated moDCs retain an immature phenotype and secrete IL-10, promoting immune evasion and the establishment of chronic infection [36,185,186,187]. Additionally, HCV from patient sera inhibits autophagy in differentiating monocytes, reducing CtsB and CtsD expression and proteolytic activity. This blocks DC maturation and antigen processing, impairing T cell responses, facilitating viral persistence, chronic hepatitis, and increasing hepatocarcinoma risk [188].

Human CMV (HCMV), a herpesvirus, can infect various DC subsets, including mDCs, moDCs, and pDCs, altering their functions and leading to virus-induced immunosuppression in patients with HCMV infection [189]. HCMV impairs antigen presentation in DCs and Langerhans cells (LCs) by targeting both MHC class II expression and endocytic protease activity. In HCMV-infected moDCs, MHC II biosynthesis is delayed and reduced, coinciding with downregulated mRNA and protein levels of cathepsins S, H, Z, B, L, and AEP, and decreased proteolytic degradation of peptide substrates [68]. Similarly, CMV lowers surface HLA-DR expression in LCs and disrupts DC maturation [69]. It has been shown that the decreased surface expression of HLA-DR in infected myeloid cells results from reduced HLA-DR transcripts, driven by lowered expression of the class II transactivator (CIITA) [70]. Additionally, HCMV encodes immunoevasins such as US2, US3, and UL83 (pp65) that interfere with MHC II; UL83 especially can alter the subcellular localization of MHC-II molecules, redirecting them to lysosomes for degradation [71]. HCMV also alters cytoskeletal architecture, affecting tubulin and fascin, leading to loss of dendritic extensions and impaired migration [69,72,73]. Therefore, cathepsins can be exploited by HCMV to dampen their inhibitory effect on antigen processing/presentation and T cell activation, thereby promoting viral immune evasion.

In the context of HIV infection, reduced cathepsin expression in DCs not only impairs viral degradation and sensing but also profoundly affects their adaptive immune functions. Decreased cathepsin activity (including cathepsins B, C, H, and Z) observed in infected moDCs impairs MHC class II antigen processing and cross-presentation, facilitating viral survival and inhibiting activation of HIV-specific T cells [78,158]. It has been shown that the viral Nef protein is responsible for decreasing MHC class I and class II expression, as well as promoting the degradation of MHC molecules through endocytosis [79]. Interestingly, HIV protease inhibitors (PIs), prescribed to HIV-infected patients, not only block the HIV aspartyl protease, preventing Gag-Pol cleavage and virion maturation, but also modulate cathepsin activity in DCs, macrophages, and CD4 T cells [80]. In DCs, ritonavir (RTV) decreases cathepsins S, D, and E, whereas nelfinavir (NFV) reduces CtsS and increases CtsD activity. These alterations affect endolysosomal antigen degradation, epitope generation, and the self MHC-peptidome, impairing antigen cross-presentation and T cell-mediated killing [78]. Collectively, these findings reinforce the central role of lysosomal proteases in shaping DC adaptive immunity and highlight their potential as therapeutic targets in HIV-1 infections [91].

CtsB in DCs plays a key role in the adaptive immune response to IAV. In BALB/c mice infected with a laboratory strain A/HKx31(H3N2), lung DCs showed increased CtsB levels and activity 30 days post-infection. Freshly isolated DCs and BMDCs up-regulate active CtsB after IAV exposure, which is required for generating T cell epitopes from exogenous ovalbumin [104]. CtsL is also essential for DC-mediated adaptive immunity against IAV and mice protection from the severe consequences of infection. *Ctsl^−/−^* mice infected with A/Puerto Rico/8/34(H1N1) showed higher lung viral loads, increased mortality, reduced CD4^+^ T cells, and impaired pathogen-specific IgG production, highlighting deficits in T cell activation and antiviral memory [81]. In contrast, ECTV infection of BMDCs silenced for CtsB, L, or S did not affect cytokine production, maturation, or CD4^+^ T cell stimulation, indicating that modulation of DC function by ECTV occurs largely independently of cathepsins [89]. This underscores that different viruses employ distinct strategies to manipulate DC-mediated immunity.

## 4. Conclusions

Cathepsins are key proteolytic enzymes that play a central role in regulating the immunobiology of DCs during viral infections. Their tightly controlled activity governs essential DC functions such as antigen uptake, endosomal processing, and peptide loading onto MHC molecules, ultimately directing T-cell priming and polarization. By coordinating both innate and adaptive immune responses, cathepsins bridge the initial, non-specific recognition of viruses and the subsequent activation of virus-specific immunity. However, many viruses have developed strategies to exploit cathepsin-dependent pathways to promote their own entry, replication, and evasion of host defenses. In this way, cathepsins occupy a dual role, firstly, acting not only as mediators of antiviral protection and secondly, as facilitators of viral pathogenesis when hijacked by pathogens.

Despite substantial progress in understanding virus-cathepsin interactions in DCs, major knowledge gaps remain regarding the subset-specific expression, regulation, and functional importance of individual cathepsins in primary human and murine DCs under physiological and pathogenic conditions. Furthermore, the molecular mechanisms by which viral infection alters cathepsin localization, expression, and enzymatic activity in different DC subsets are insufficiently characterized. Therefore, future studies are needed to define the specific contribution of individual cathepsins in regulating antiviral functions in different subsets of DCs.

Therapeutically, selective modulation of cathepsin activity represents a promising strategy to fine-tune antiviral immunity. Targeting cathepsins is a promising antiviral strategy, as these proteases are essential for viral entry and processing. Synthetic inhibitors, such as K11777, MDL 28170, Z LVG CHN2, and ONO 5334, block cathepsin-mediated viral entry and protein processing, often showing enhanced efficacy when combined with serine protease inhibitors. Natural compounds, including E-64, gallinamide A, tokaramide A, miraziridine A, and aloperine, selectively inhibit cathepsins B or L, preventing viral assembly, entry, and propagation in diverse viruses, including SARS-CoV-2, EBOV, HIV-1, HCV, IAV, and MERS-CoV. These findings underscore cathepsin inhibition as a versatile therapeutic approach to limit viral infection and spread [176]. The interplay between cathepsins and their endogenous inhibitors, such as cystatins, also provides opportunities to restore proteolytic homeostasis during infection or inflammation. A comprehensive understanding of cathepsin-mediated regulation will not only elucidate the mechanisms underlying viral immune evasion but also inform the development of novel immunotherapeutic and antiviral interventions targeting DCs.

## Figures and Tables

**Table 1:** Cathepsin mRNA expression profiles across dendritic cell subsets.

Cathepsin	cDC1	cDC2	migDC	pDC	moDC	Refs.
A (serine)	moderate to high	moderate to high	ND	moderate	moderate to high	[11,30]
B (cysteine)	moderate	moderate to high	ND	high	moderate to high	[18,19,20,24]
C (cysteine)	low to moderate	moderate	ND	high	moderate	[24,27,29]
D (aspartic)	moderate	moderate	ND	moderate to high	moderate	[11,24,31]
E (aspartic)	ND	ND	ND	low	low to moderate	[24,26]
F (cysteine)	ND	ND	ND	very low	ND	[24]
G (serine)	low	low	ND	low to moderate	low	[11,30]
H (cysteine)	low to moderate	moderate to high	ND	low	moderate	[23,25]
K (cysteine)	ND	ND	ND	low	ND	[24]
L (cysteine)	moderate	high	ND	high	high	[18,19,20]
O (cysteine)	ND	ND	ND	low	ND	[24]
S (cysteine)	moderate	high	low	moderate	very high	[18,19,20,24]
V (cysteine)	ND	ND	ND	moderate to high	ND	[24]
X(Z/P) (cysteine)	moderate to high	high	ND	low	high	[21,22,24,25,27,28]
W (cysteine) *	ND	ND	ND	ND	ND	-

* almost exclusively expressed in natural killer (NK) cells and cytotoxic T lymphocytes (CTLs). cDC, conventional dendritic cells; migDC, migratory DC; pDC, plasmacytoid DC; moDC, monocyte-derived DC; ND, not determined; NK, natural killer cell; CTL, cytotoxic T lymphocyte.

## Data Availability

No new data were created or analyzed in this study.

## Abbreviations

The following abbreviations are used in this manuscript:

ACE2	angiotensin-converting enzyme
AEP	asparaginyl endopeptidase
APC	antigen-presenting cell
BMDC	bone marrow-derived dendritic cell
CD-MPR	cation-dependent mannose-6-phosphate receptor
cGAS	cyclic GMP-AMP synthetase
CLIP	class II-associated invariant chain peptide
CMV	cytomegalovirus
CTL	cytotoxic T lymphocyte
DC	dendritic cell
EBOV	Ebola virus
ECTV	ectromelia virus
FLT3	tyrosine kinase 3 ligand receptor
HCMV	human cytomegalovirus
HCV	hepatitis C virus
HeV	Hendra virus
HIV-1	human immunodeficiency virus 1
IAV	influenza A virus
IFN	interferon
Ii	invariant chain
IL	interleukin
IRF	interferon regulatory factor
MAVS	mitochondrial antiviral signaling protein
MHC	major histocompatibility complex class
moDCs	monocyte-derived DCs
MyD88	myeloid differentiation primary response 88
NiV	Nipah virus
NLR	nucleotide-binding oligomerization domain (NOD)-like receptors
NPC1	Niemann-Pick type C1 protein
pDC	plasmacytoid dendritic cell
PI	protease inhibitor
PPARγ	peroxisome proliferator-activated receptor γ
PRR	pattern recognition receptor
rER	rough endoplasmic reticulum
RIG-I	retinoic acid-inducible gene-I
RLR	RIG-I-like receptor
SARS-CoV	severe acute respiratory syndrome coronavirus
STING	stimulator of interferon genes
Tfh	T follicular helper cell
TGN	trans-Golgi network
TIR	Toll/IL 1 receptor
TLR	Toll-like receptor
TRIF	TIR-domain-containing adapter-inducing interferon-β
VLP	viral-like particle
vRNP	viral ribonucleoprotein
